# Integrative single-cell analysis of LUAD: elucidating immune cell dynamics and prognostic modeling based on exhausted CD8+ T cells

**DOI:** 10.3389/fimmu.2024.1366096

**Published:** 2024-03-26

**Authors:** Han Zhang, Pengpeng Zhang, Xuefeng Lin, Lin Tan, Yuhang Wang, Xiaoteng Jia, Kai Wang, Xin Li, Daqiang Sun

**Affiliations:** ^1^ Clinical School of Thoracic, Tianjin Medical University, Tianjin, China; ^2^ Department of Lung Cancer Surgery, Tianjin Medical University Cancer Institute and Hospital, Tianjin, China; ^3^ Tianjin Medical College, Tianjin, China; ^4^ Qingdao Hospital, University of Health and Rehabilitation Sciences, Qingdao Municipal Hospital, Qingdao, China; ^5^ Tianjin Chest Hospital, Tianjin University, Tianjin, China

**Keywords:** LUAD, myeloid, CD4+ T cells, CD8+ T cells, pDCs, GALNT2

## Abstract

**Background:**

The tumor microenvironment (TME) plays a pivotal role in the progression and metastasis of lung adenocarcinoma (LUAD). However, the detailed characteristics of LUAD and its associated microenvironment are yet to be extensively explored. This study aims to delineate a comprehensive profile of the immune cells within the LUAD microenvironment, including CD8+ T cells, CD4+ T cells, and myeloid cells. Subsequently, based on marker genes of exhausted CD8+ T cells, we aim to establish a prognostic model for LUAD.

**Method:**

Utilizing the Seurat and Scanpy packages, we successfully constructed an immune microenvironment atlas for LUAD. The Monocle3 and PAGA algorithms were employed for pseudotime analysis, pySCENIC for transcription factor analysis, and CellChat for analyzing intercellular communication. Following this, a prognostic model for LUAD was developed, based on the marker genes of exhausted CD8+ T cells, enabling effective risk stratification in LUAD patients. Our study included a thorough analysis to identify differences in TME, mutation landscape, and enrichment across varying risk groups. Moreover, by integrating risk scores with clinical features, we developed a new nomogram. The expression of model genes was validated via RT-PCR, and a series of cellular experiments were conducted, elucidating the potential oncogenic mechanisms of GALNT2.

**Results:**

Our study developed a single-cell atlas for LUAD from scRNA-seq data of 19 patients, examining crucial immune cells in LUAD’s microenvironment. We underscored pDCs’ role in antigen processing and established a Cox regression model based on CD8_Tex-LAYN genes for risk assessment. Additionally, we contrasted prognosis and tumor environments across risk groups, constructed a new nomogram integrating clinical features, validated the expression of model genes via RT-PCR, and confirmed GALNT2’s function in LUAD through cellular experiments, thereby enhancing our understanding and approach to LUAD treatment.

**Conclusion:**

The creation of a LUAD single-cell atlas in our study offered new insights into its tumor microenvironment and immune cell interactions, highlighting the importance of key genes associated with exhausted CD8+ T cells. These discoveries have enabled the development of an effective prognostic model for LUAD and identified GALNT2 as a potential therapeutic target, significantly contributing to the improvement of LUAD diagnosis and treatment strategies.

## 1 Introduction

In the past decade, LUAD has emerged as the predominant subtype of lung cancer, accounting for approximately 40% of all lung cancer cases (1, 2). The incidence of this form of lung cancer is not only increasing among chronic smokers but also shows an upward trend in former and even non-smokers. Despite significant advancements in treatment modalities such as the introduction of PD-L1-targeted immunotherapies (3, 4) and targeted therapies for specific genetic mutations like KRAS and EGFR (5, 6), LUAD continues to pose a major public health challenge globally. The five-year survival rate for this malignancy varies based on region, treatment accessibility, and individual differences, generally remaining low, typically ranging from 5% to 20% (7).

TME refers to the environment surrounding tumor cells, encompassing nearby blood vessels, immune cells, fibroblasts, the extracellular matrix, and secreted molecules (8). It plays a critical role in tumor development, metastasis, and response to treatment. In LUAD, characteristics of the TME such as immune cell infiltration, local inflammatory responses, and interactions between tumor cells and their surrounding environment are key factors affecting disease progression and treatment efficacy (9). Increasing research emphasizes the significance of the TME in predicting cancer prognosis and responses to immunotherapy (10, 11). Understanding these aspects not only aids in deepening our comprehension of the biological characteristics of LUAD but also may facilitate the development of new treatment strategies, such as the use of immunomodulatory drugs or therapies targeting specific features of the TME.

Single-cell RNA sequencing (scRNA-seq) represents a significant advancement over conventional bulk RNA-sequencing (RNA-seq) by offering detailed transcriptomic analysis at the individual cell level (12). This advanced methodology has been transformative in tumor microenvironment research, yielding high-resolution insights across various cancers. Studies in malignancies such as lymphoma, melanoma, and liver cancer have utilized scRNA-seq to uncover complex details regarding immune cell heterogeneity, composition, and regulatory mechanisms within their respective tumor microenvironments (13–15). Despite these advancements, the exploration of the LUAD tumor microenvironment using scRNA-seq is still in its nascent stage, underscoring an urgent need for more in-depth studies in this area to enhance our understanding.

In this study, our objective was to construct a comprehensive single-cell atlas of LUAD by performing scRNA-seq on 29 samples collected from 19 LUAD patients (16). Utilizing this atlas, we meticulously analyzed the canonical immune cell types within the LUAD microenvironment, with a focus on myeloid cells, CD4+ T cells, and CD8+ T cells. We identified plasmacytoid dendritic cells (pDCs) as key regulators in antigen processing and presentation via the MHC-II signaling pathway. By integrating scRNA-seq data with bulk RNA-seq data from LUAD patients, we successfully identified a set of risk genes based on CD8_Tex-LAYN (exhausted CD8+ T cells) marker genes. Subsequently, we developed a robust and reliable Cox regression model that accurately assesses the risk levels of LUAD patients. Based on risk stratification, we systematically evaluated differences in prognosis, tumor microenvironment, and mutation landscape between high-risk and low-risk groups. Furthermore, combining clinical characteristics and risk scores, we constructed a novel nomogram. Lastly, we validated the expression of model genes via RT-PCR and determined GALNT2 as a potential therapeutic target for LUAD through a series of cellular experiments. These findings not only enhance our understanding of the LUAD microenvironment but also offer promising prospects for improving diagnostics and prognosis in LUAD clinical settings.

## 2 Methods

### 2.1 Data collection of LUAD singel-cell datasets

The original scRNA-seq data used in this study were obtained from 19 treatment-naïve LUAD patients (16), including primary lung tumors (tLung, n = 11), distant normal lung tissues (nLung, n = 11), and lymph node metastases (mLN, n = 7). These data were acquired from the the Gene Expression Omnibus (GEO) database under the accession number GSE131907. The training data for our prognostic model, including the gene expression matrix(FPKM format), clinical specifics, and mutation details for LUAD patients, were directly downloaded from The Cancer Genome Atlas (TCGA) via the following portal: https://portal.gdc.cancer.gov/repository. Additionally, the external validation cohorts, comprising gene expression and clinical data (GSE31210, GSE37745, GSE50081, GSE68465, GSE3141), were acquired from the GEO database to assess the model’s predictive accuracy. To make the data from TCGA and GEO more compatible, we converted the format from FPKM to TPM and then used the R package ‘SVA’ to correct for batch effects between the datasets.

### 2.2 Evaluating data from single-cell RNA sequencing

The gene-cell matrix for each sample was individually imported to Scanpy (version 1.9.1) for downstream analysis. Cells with the unusual number of UMIs (≥ 8,000), number of detected genes (≤ 500 or ≥ 4,000) or mitochondrial gene percent (≥ 10%) were excluded. The UMI count for the genes in each cell was normalized by the “LogNormalize” method as the following formula:

The gene-cell matrix for each sample was imported into Scanpy (version 1.9.1) (17) for downstream analysis. To ensure data quality, cells with unusual characteristics were excluded from the analysis. These characteristics included an abnormal number of UMIs (≥ 8,000), a low number of detected genes (≤ 500) or a high number of detected genes (≥ 4,000), as well as a high percentage of mitochondrial genes (≥ 10%). Subsequently, the UMI count for each gene in every cell was normalized using the “LogNormalize” method as the following formula:


$$Gene\;A\;expression\;level=\log\left(1+\frac{UMI_{A}}{UMI_{Total}}\times 10^{5}\right)$$

To alleviate the impact of batch effects during the clustering process, we utilized Harmony to integrate all the samples. Specifically, we identified 2,000 highly variable genes in each sample using a variance stabilizing transformation. Next, we determined anchors between the individual datasets and computed correction vectors to create an integrated expression matrix. This integrated matrix was then utilized for subsequent cell clustering, enhancing the robustness and reliability of the analysis by effectively addressing batch effects. The integrated expression matrix was utilized to calculate the principal components (PCs). From these PCs, a subset of significant ones was selected, and the cells were categorized into sub-clusters using the Louvain algorithm. To visualize the clustering results, we employed the Uniform Manifold Approximation and Projection (UMAP) technique. The cell type annotations for each cluster were determined based on the expression patterns of known marker genes using CellTypist (https://github.com/Teichlab/celltypist). The expression of each gene in a given cluster was compared to the rest of the cells using the Wilcoxon rank sum test. Genes meeting the following criteria were considered as significantly upregulated: Firstly, they exhibited a log2(foldchange) value ≥ 1 or ≤ -1, indicating substantial overexpression in the target cluster. Secondly, these genes were expressed in more than 25% of the cells within the target cluster. Finally, the adjusted p-value for each gene was required to be less than 0.05, demonstrating statistical significance in the differential expression analysis. Monocle3 R package (https://cole-trapnell-lab.github.io/monocle3/) was used for pseudotime trajectory analysis (18).

### 2.3 Cell-cell communication analysis

We employed the CellChat toolkit (https://github.com/sqjin/CellChat) within the R programming environment (19). This analysis aimed to elucidate the differential interactions and signaling pathways among various cell types, including DC1, DC2, pDC, Monocytes, Migratory DCs, and Mast cells. CellChat, a cutting-edge tool, enables the quantitative inference of intercellular communication networks from scRNA-seq data. It leverages a comprehensive database of human ligand-receptor interactions and advanced pattern recognition techniques to predict the primary signaling mechanisms among cells, thereby illuminating the coordination of cellular functions. To ensure the analysis’s relevance and accuracy, only ligand-receptor pairs exhibiting a P-value less than 0.05 were considered, allowing for a focused evaluation of the intricate relationships between diverse cell types.

### 2.4 Simultaneous gene regulatory network analysis

To assess the transcriptional distinctions among cell clusters (CD8_Tn-LEF1, CD8_Tem-GZMK, CD8_Trm-KLRB1, and CD8_Tex-LAYN), based on transcription factors and their target genes, we conducted pySCENIC (version 0.10.0) analysis on all single cells. This approach facilitated the identification of regulons preferentially expressed within these clusters, employing the Limma package for calculation. Our analysis focused solely on regulons that exhibited significant upregulation or downregulation in at least one of the clusters, considering only those with an adjusted p-value of less than 0.05 for further exploration.

### 2.5 Construction and validation of prognostic signature

The prognostic relevance of CD8_Tex-LAYN and CD8_Tn-LEF1 as cellular biomarkers for overall survival in patients with lung adenocarcinoma (LUAD) from the TCGA database was initially evaluated through univariate Cox regression, pinpointing genes of prognostic significance at a threshold of p< 0.01. Subsequently, we refined our prognostic gene assessment using the “glmnet” R package (20) to implement a LASSO Cox proportional hazards model. Further refinement was achieved by integrating this model with a multivariate Cox regression analysis, thereby constructing a risk model that incorporates gene expression levels weighted by their associated risk coefficients. This composite approach yielded six principal genes warranting additional prognostic scrutiny. To differentiate between high and low-risk patient cohorts, a median value cutoff was applied. The survival outcomes for these cohorts within the TCGA-LUAD dataset and across five independent GEO datasets (GSE3141, GSE50081, GSE68465, GSE37745, and GSE31210) were analyzed using Kaplan-Meier survival curves, facilitated by the “survival” and “survminer” R packages. The prognostic potency of the derived risk score was corroborated by generating ROC curves and computing the AUC with the “survivalROC” R package (21), thereby quantifying the risk model’s predictive precision.

### 2.6 Nomogram development

In developing the nomogram, we first executed both univariate and multivariate Cox regression analyses on the risk scores and clinical characteristics of patients with lung adenocarcinoma (LUAD) from the TCGA database. The objective was to pinpoint variables that held significant prognostic weight for LUAD. Following this, using the ‘rms’ R package (22), we constructed the final nomogram. To evaluate its effectiveness, we employed calibration curves and decision curve analysis (DCA).

### 2.7 Mutation analysis

For the analysis of mutation data and clinical details, we utilized the ‘maftools’ R package. The function read.maf was applied to import this information into a maf file format. We then employed plotmafSummary to examine the mutation profile of patients with LUAD in the TCGA dataset. For visualizing the mutation characteristics in both high and low-risk groups, the oncoplot function was used to create heatmaps that integrated clinical data with mutation information. To investigate co-mutation patterns among key genes and the top 10 most frequently altered genes in TCGA-LUAD, the somaticInteractions function was deployed. Tumor Mutational Burden (TMB) refers to the total number of non-synonymous mutations within the genome of tumor cells. We downloaded the maf files for LUAD from the TCGA database, then assessed the TMB levels for each patient. Subsequently, we applied a logarithmic transformation to reduce data skewness and minimize the impact of outliers on the analysis results. Finally, we compared the differences in TMB between the high-risk and low-risk groups, and calculated the correlation between the risk score and TMB.

### 2.8 Enrichment analysis

Our study commenced with the implementation of the ‘GSVA’ algorithm (23), aiming to identify Hallmarker pathways that were predominantly enriched in the high-risk group compared to the low-risk group. Following this, we employed the ‘GSEA’ algorithm (24) to distinguish and analyze pivotal Kyoto Encyclopedia of Genes and Genomes Pathways (KEGG) (25) in both high and low-risk groups. The ssGSEA algorithm (26) played a crucial role in evaluating the correlation between enrichment scores, tumor immunity cycle, and tumor-related pathways. It also proved instrumental in measuring variances in immune cell types and immune functions across the high and low-risk categories.

### 2.9 Assessment of the tumor microenvironment

We procured data regarding immune cell infiltration from seven different databases via the Timer2.0 platform, accessible at (http://timer.comp-genomics.org/) (27).Subsequently, we conducted an analysis to compare the levels of immune cell infiltration between high and low-risk groups. Utilizing the ‘estimate’ R package (28), we calculated the stromal, immune, and TumorPurity scores, along with the ESTIMATE scores for each specimen in the TCGA-LUAD dataset.

### 2.10 Immunotherapy response evaluation

Differences in the expression of immune checkpoint genes and Major Histocompatibility Complex (MHC) genes were compared between the high and low-risk groups. Correlations between central genes, risk scores, and these immune-related genes were also calculated. Variations in Immunophenoscoring (IPS) between the high and low-risk groups were assessed. Additionally, the potential for immune escape in these groups was evaluated using the tumor immune dysfunction and exclusion (TIDE) algorithm (29).

### 2.11 Cultivation of cell lines

In our laboratory setting, normal human lung epithelial cells (BEAS-2B) and LUAD cell lines (A549, H1299, H1975, H1650) were procured from the Cell Resource Center at the Shanghai Institute for Biological Sciences. These cells were cultured in RPMI-1640 medium, produced by Gibco BRL, USA, supplemented with 10% fetal bovine serum (FBS) obtained from Cell-Box, Hong Kong, and 1% penicillin-streptomycin solution, supplied by Biosharp, China. Cultivation of these cells was carried out in a controlled environment, maintained at 37°C with 5% CO2 and 95% humidity.

### 2.12 RNA extraction and reverse transcription PCR analysis

Total RNA was isolated from the cell lines using the TRIzol reagent (15596018, Thermo Fisher Scientific), adhering to the instructions provided by the manufacturer. The PrimeScriptTM RT kit (R232-01, Vazyme) was then utilized for synthesizing cDNA. This was followed by quantitative RT-PCR analysis, conducted using the SYBR Green Master Mix (Q111-02, Vazyme), with GAPDH mRNA serving as the normalization control. The relative gene expression levels were determined employing the 2−ΔΔCt method. Primers used in this study were sourced from Nanjing Sunbio Technology Co., Ltd. (Nanjing, China), detailed in **Supplementary Table 1**.

### 2.13 Migration and invasion analysis via transwell assays

Migration and invasion capacities were tested using 24-well transwell inserts, with A549 and H1299 cells seeded at 1×10^5^ cells in the upper chamber. In the invasion assays, we prepared the chambers by pre-coating them with matrigel from BD Biosciences, USA. For migration assays, however, the chambers were left uncoated. Following the migration or invasion process, cells located on the lower side of the membrane were fixed and subsequently stained using crystal violet sourced from Solarbio, China.

### 2.14 Assessment of cellular proliferation using the CCK-8 method

In this procedure, cells were plated in 96-well plates at a concentration of 3×10³ cells per well. Following cell seeding, 10 mL of CCK-8 solution (A311-01, Vazyme) was added to each well. The plates were then incubated in the dark at 37°C for 2 hours. The proliferation of cells was determined by measuring absorbance at 450 nm with a spectrophotometer (A33978, Thermo Fisher Scientific) at time points of 0, 24, 48, 72, and 96 hours.

### 2.15 Assessment of colony formation

In this part of the study, cells were seeded at a density of 1×10³ cells per well in 6-well plates, followed by a growth period of 14 days. After this incubation, cells were washed with PBS and fixed with 4% paraformaldehyde for 15 minutes. The colonies were then stained with Crystal Violet, provided by Solarbio, China.

### 2.16 Statistical methods

Bioinformatics data were analyzed using R (version 4.3.1), while Graphpad and ImageJ were employed for experimental data. Intergroup differences were evaluated using the T-test or one-way ANOVA for normal distributions, and Wilcoxon or Kruskal-Wallis tests for non-normal data. Survival was analyzed via Kaplan-Meier curves and Log-rank tests. Spearman’s correlation assessed relationships between datasets. Significance was set at p< 0.05, with *P< 0.05, **P< 0.01, ***P< 0.001 indicating varying significance levels.

## 3 Results

### 3.1 Construction of a scRNA-seq Atlas for LUAD

**Figure 1:**
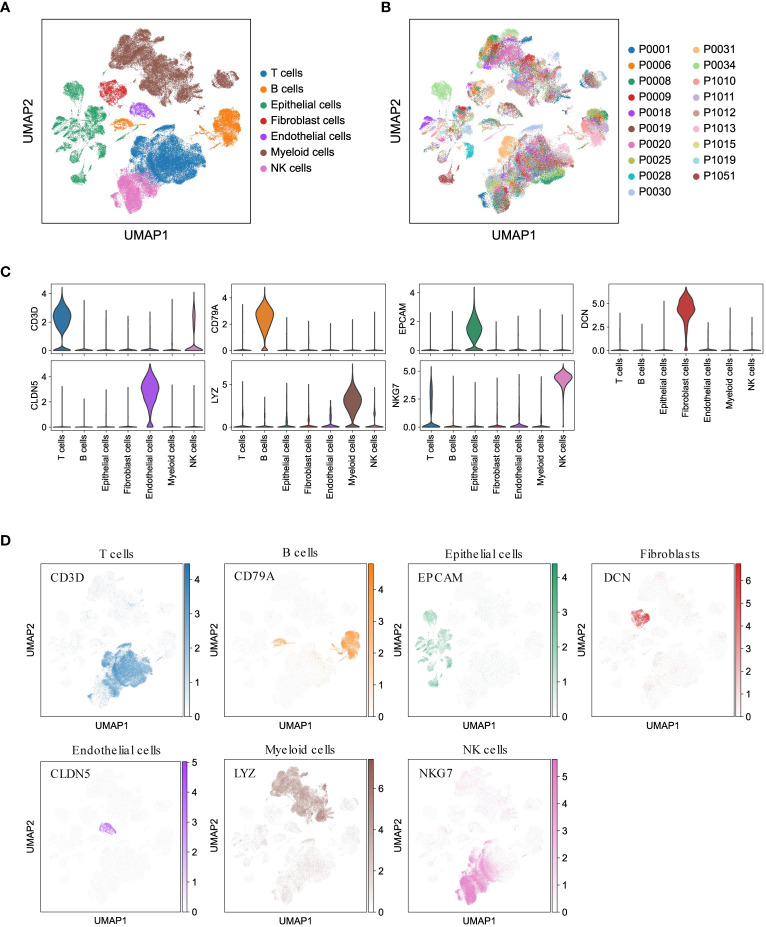
Comprehensive LUAD single-cell atlas. **(A, B)** UMAP plots of 107,751 cells from 19 patients, colored by major cell lineages and patients. **(C)** Violin plots for expression of canonical marker genes in each major cell lineage. **(D)** UMAP plots of seven major cell lineages, colored by corresponding canonical marker genes.

To gain valuable insights into the cellular composition of the tumor microenvironment in LUAD, we performed scRNA-seq analysis on 19 treatment-naïve LUAD patients. This included collecting samples from primary lung tumors (tLung, n = 11), distant normal lung tissues (nLung, n = 11), and lymph node metastases (mLN, n = 7). We carefully addressed batch effects and annotated canonical cell markers (detailed in the Methods section), enabling us to classify a total of 107,751 cells into seven major cell lineages: epithelial cells, stromal cells (including fibroblasts and endothelial cells), and immune cells (T cells, B cells, myeloid cells, and NK cells) (**Figure 1A**). Notably, the cellular composition demonstrated consistency across different patients (**Figure 1B**), further validating our analysis. As anticipated, these major cell lineages exhibited the expression of specific canonical marker genes (**Figure 1C**) and formed distinct clusters (**Figure 1D**), affirming the accuracy of our lineage classification. In summary, our integration of scRNA-seq data from 19 LUAD patients successfully led to the construction of a comprehensive single-cell atlas of LUAD. This atlas serves as a solid foundation for our subsequent analyses, which aim to delve into the cellular composition and function of the LUAD tumor microenvironment.

### 3.2 Analysis of macrophage profiles in LUAD

**Figure 2:**
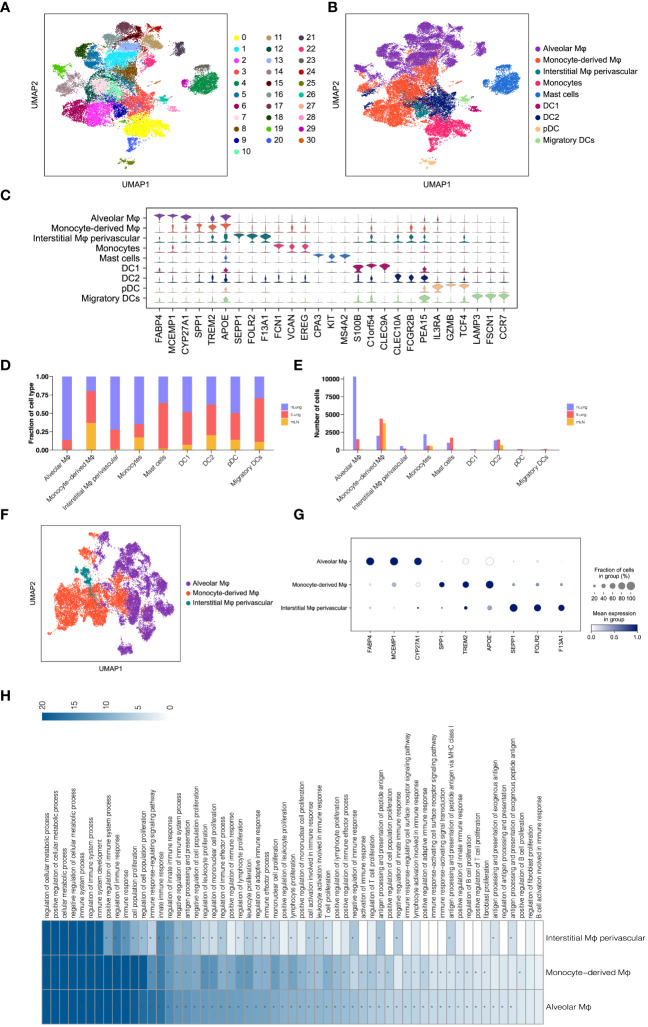
Analysis of Macrophages in LUAD. **(A, B)** UMAP of 33,561 myeloid cells, indicating clusters and cell types. **(C)** Violin plots for top threeDEGs in nine myeloid subsets. **(D, E)** Comparison of proportions and cell numbers in nine myeloid subsets across nLung, tLung, and mLN. **(F)** UMAP of 22,959 macrophages, categorized by cell types. **(G)** Dot plot of marker gene expression in three macrophage subsets. **(H)** KEGG pathway analysis results for three macrophage subsets.

In addition to T lymphocytes, the significance of myeloid cells as crucial components of tumor-infiltrating cells and regulators of tumor inflammation and angiogenesis has been emphasized in numerous studies (30, 31). To gain insights into the subsets of myeloid cells in LUAD, we categorized a total of 33,561 myeloid cells into 31 sub-clusters using the Louvain algorithm (resolution = 1.0) (**Figure 2A**). These sub-clusters were further assigned to nine major subsets based on canonical cell markers (**Figure 2B**). The major myeloid subsets identified include macrophages (alveolar Mφ, monocyte-derived Mφ, and interstitial Mφ perivascular), monocytes, mast cells, and dendritic cells (DC1, DC2, pDC, and migratory DCs). The expression of gene signatures specific to each subset confirmed the accuracy of our cell annotation (**Figure 2C**). These findings contribute to the characterization of the diverse myeloid cell populations within the LUAD tumor microenvironment, providing a foundation for further understanding their roles in tumor progression and immunomodulation.

Subsequently, we examined the relative proportion and cell numbers of these myeloid cell subsets across different tissues. Interestingly, macrophages exhibited significant variations both in proportion (**Figure 2D**) and cell numbers (**Figure 2E**) compared to other cell lineages. To ensure the reliability and validity of our analysis, we specifically focused on macrophages as a distinct population within the total myeloid cell population, in order to explore their unique properties and functions. By re-clustering the 22,959 macrophages using UMAP (**Figure 2F**) analyses, we identified three distinct macrophage subsets: alveolar Mφ, monocyte-derived Mφ, and interstitial Mφ perivascular. Each of these subsets exhibited unique gene signatures (**Figure 2G**). Furthermore, KEGG pathway analysis revealed that these tumor-associated macrophages (TAMs) in LUAD primarily participate in immune regulation, cell proliferation, and antigen processing and presentation (**Figure 2H**). These findings are consistent with the well-established role of macrophages in shaping the tumor microenvironment and engaging in tumor immunity (32, 33). By investigating the distinct subsets and functions of macrophages within the LUAD tumor microenvironment, we gain a better understanding of their contributions to tumor progression and immune responses. This knowledge holds potential for targeted therapeutic strategies aimed at modulating macrophage-mediated immune regulation in LUAD.

### 3.3 Analysis of monocytes, mast cells, and dendritic cells in LUAD

**Figure 3:**
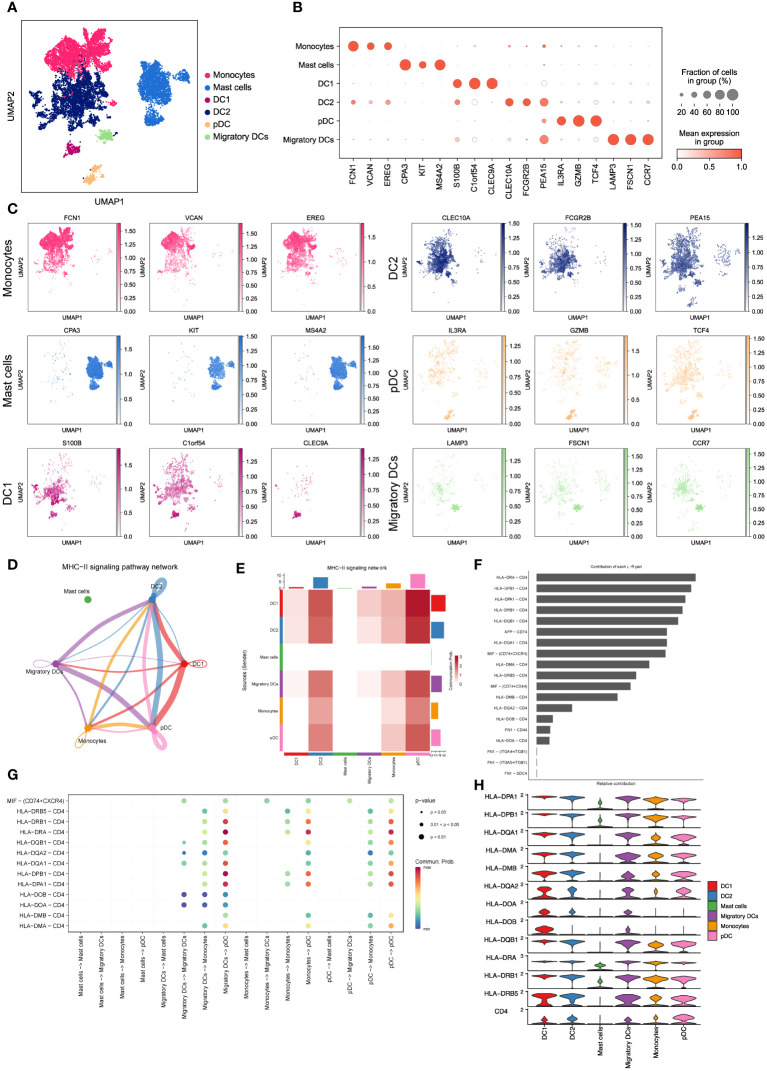
Characterizing monocytes, mast cells and dendritic cells in LUAD. **(A)** UMAP plot of 10,602 myeloid cells, including monocytes, mast cells and dendritic cells. **(B)** Dot plot for expression of top3 DEGs in six myeloid cell subsets. **(C)** UMAP plots of six myeloid cell subsets, colored by corresponding marker genes. **(D)** The inferred MHC-II signaling network by Cell-Chat. The edge width represents the communication probability. **(E)** Heatmap of the communication probability among six myeloid cell subsets in MHC-II signaling pathway. **(F)** Relative contributions of 19 ligand-receptor pairs to the overall communication network of MHC-II signaling pathway. **(G)** Significant ligand-receptor pairs that contribute to MHC-II signaling among the mast cell, migratory DC, monocyte and pDC. **(H)** Violin plot for expression of canonical MHC-II signaling genes in six myeloid cell subsets.

To gain further insights into the properties and functions of myeloid cells other than macrophages, we employed UMAP and tSNE methods to re-cluster the remaining 10,602 myeloid cells. This analysis resulted in the identification of seven subsets, including monocytes, mast cells, and four types of dendritic cells (DC1, DC2, pDC, and migratory DCs) (**Figure 3A** and **Supplementary Figure 1A**). As expected, each subset displayed specific enrichment of corresponding cell marker genes (**Figures 3B, C**, **Supplementary Figures 1B, C**), providing strong evidence for the accuracy of our cell type annotation. Next, we employed CellChat analysis (19) to explore the intercellular communication network among these myeloid cell subsets. Intriguingly, we observed potential intercellular communication in the MHC-II signaling pathway network among monocytes, DC1, DC2, pDC, and migratory DCs (**Figure 3D**). Specifically, DC1, DC2, and pDC exhibited close connections within the MHC-II signaling pathway (**Figure 3E**). This finding suggests possible interactions and shared functionalities among these subsets, supported by their similar gene expression patterns (**Supplementary Figure 1D**) and distribution within LUAD tissues (**Figures 2D, E**). These results shed light on the intricate interplay and communication networks existing among myeloid cell subsets in the LUAD tumor microenvironment. Understanding these interactions provides valuable insights into the regulatory mechanisms and potential therapeutic targets associated with myeloid cells in LUAD.

To validate the significance of activated MHC-II signaling in LUAD, we examined the contributions of 19 curated ligand-receptor pairs to the MHC-II signaling pathway network. As depicted in **Figure 3F**, the HLA-DRA-CD4 pair emerged as a prominent contributor to this communication network, underscoring the pivotal role of antigen presentation in activated MHC-II signaling in LUAD. Moreover, our analysis predicted pDCs to be the predominant cell type in the MHC-II signaling pathway network (**Figure 3G**). Notably, pDCs exhibited high expression of MHC-II signaling marker genes (**Figure 3H**), aligning with their crucial function in tumor antigen presentation during tumor immunity (19, 34). Furthermore, we have discovered that these myeloid cells engage in intercellular communication via the macrophage migration inhibitory factor (MIF) signaling pathway (**Supplementary Figures 1E, F**). This observation suggests a critical involvement of hypoxia in regulating the LUAD microenvironment and influencing metastatic processes (35). These findings collectively demonstrate that dendritic cells, particularly pDCs, heavily rely on antigen processing and presentation in the MHC-II signaling pathway to regulate LUAD progression. This highlights the significance of antigen presentation and the potential involvement of pDCs in shaping the tumor microenvironment and immune responses in LUAD.

### 3.4 Profiling diverse CD4+ T cell states in LUAD

**Figure 4:**
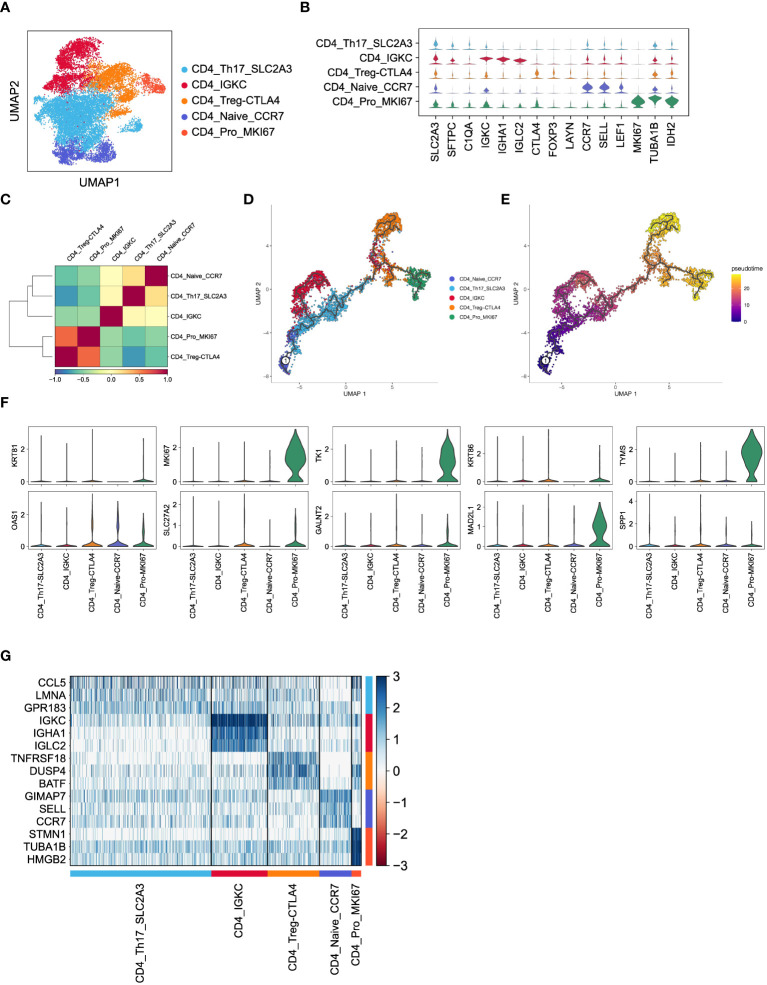
Inferred CD4+ T cell states in LUAD patients. **(A)** UMAP plot of 15,711 CD4+ T cells, colored by cell types. **(B)** Violin plot for expression of canonical cell-type marker genes in five major CD4+ T cell subsets. **(C)** Correlations of gene expression among five major CD4+ T cell subsets. **(D, E)** Pseudotime analysis for profiling trajectory of differentiating CD4+ T cells, colored by cell types and pseudotime. **(F)** Violin plots for expression of ten CD8_Tex-LAYN signatures in five major CD4+ T cell subsets. **(G)** Heatmap of top3 DEGs in five major CD4+ T cell subsets.

To investigate the functions of CD4+ T cells in LUAD, we categorized a total of 15,711 CD4+ T cells into five major subsets based on marker gene expression (**Figure 4A**). Specifically, the CD4_Th17_SLC2A3 subset displayed an enrichment of genes related to T helper 17 (Th17) cells, such as SLC2A3, SFTPC, and C1QA. The CD4_IGKC subset exhibited high expression of immunoglobulin-related genes, including IGKC, IHA1, and IGLC2. The CD4_Treg_CTLA4 subset demonstrated elevated expression of inhibitory receptors like CTLA4, FOXP3, and LAYN, representing CD4+ regulatory T cells (Tregs). The CD4_Naive_CCR7 subset displayed higher expression of genes associated with naïve CD4+ cells, such as CCR7, SELL, and LEAF1. Lastly, the CD4_Pro_MKI67 subset showed an enrichment of proliferation markers, including MKI67, TUBA1B, and IDH2, indicating the presence of proliferating cells (**Figure 4B**). As expected, these subsets exhibited distinct gene expression patterns (**Figure 4G**), which reflect their diverse functions and regulatory roles within LUAD.

**Figure 5:**
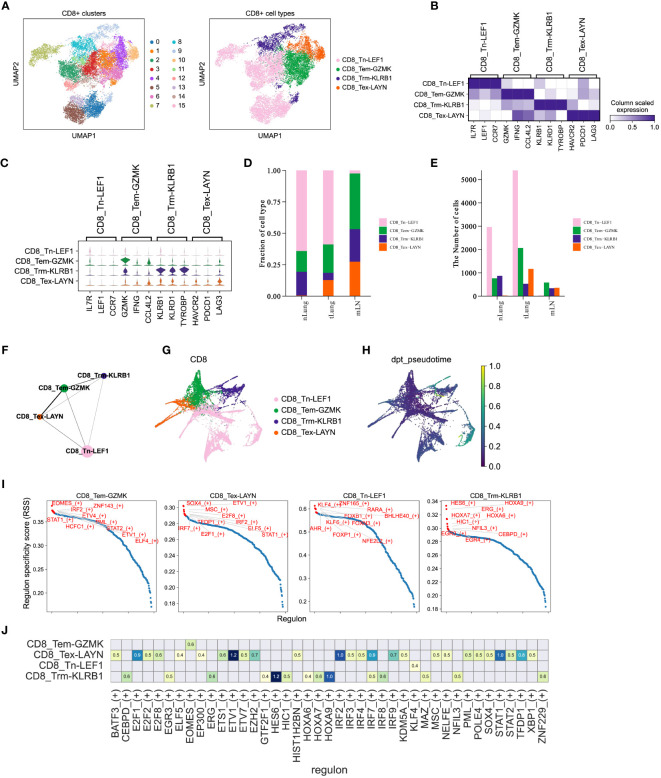
CD8+ T cells mediate immune responses during LUAD progression. **(A)** UMAP plot of 15,092 CD8+ T cells, colored by clusters (left) and cell types (right). **(B, C)** Matrixplot and violin plot for expression of canonical cell-type marker genes in four major CD8+ T cell subsets. **(D, E)** Proportions and cell numbers of four major CD8+ T cell subsets across nLung, tLung and mLN tissues. **(F, G)** PAGA graph of four major CD8+ T cell subsets at cell-type and single-cell resolution. **(H)** Pseudotime analysis for profiling trajectory of differentiating CD8+ T cells. **(I)** Regulon specificity score of curated transcription factors in four major CD8+ T cell subsets. The top 10 transcription factors with the highest scores were colored in red. **(J)** Activities of immune-relevant transcription factors in our major CD8+ T cell subsets.

Subsequently, we conducted pairwise correlation analysis of gene expression levels among the five CD4+ T cell subsets. Interestingly, we observed a relatively higher correlation between the CD4_Pro_MKI67 and CD4_Treg_CTLA4 subsets (**Figure 4C**), which is consistent with their known immunosuppressive roles in non-small cell lung cancer (36, 37). Furthermore, pseudotime analysis of these CD4+ T cell subsets revealed a clear transition trend from a naïve state to an exhausted state (**Figures 4D, E**), indicating an ongoing process of immune exhaustion during the progression of LUAD (16). Importantly, we found that ten upregulated genes (**Figure 5A**) in the previously mentioned CD8_Tex-LAYN subset, particularly MKI67, TK1, MAD2L1, and TYMS, were also highly expressed in the CD4_Pro_MKI67 subset (**Figure 4F**). This implies a close collaboration between exhausted CD4+ and CD8+ T cells in promoting tumor immunosuppression (38) Taken together, these findings indicate that CD4+ T cells can differentiate into distinct states with specific gene expression patterns, and they work in concert with CD8+ T cells to regulate tumor progression in LUAD.

### 3.5 Profiling diverse CD8+ T cell states in LUAD

CD8+ T cells are widely recognized as crucial contributors to anti-tumor immunity across different cancer types (13, 39, 40) and have been closely linked to immunotherapy response (14, 41, 42). In order to enhance our understanding of the CD8+ T cell composition in LUAD, we utilized the Louvain algorithm in Scanpy to classify a total of 15,092 CD8+ T cells into 16 sub-clusters (**Figure 5A**, left panel). Subsequently, based on the expression of marker genes, we further categorized these cells into four major subsets (**Figure 5A**, right panel). In **Figures 5B, C**, we displayed the expression of marker genes for each cell type. Importantly, the proportions (**Figure 5D**) and cell numbers (**Figure 5E**) of these four major CD8+ T cell subsets exhibited variations across different tissues, aligning with the complex and dynamic composition of T cells in LUAD progression and metastasis. To gain a deeper understanding of the characteristics of distinct CD8+ T cell subsets in LUAD, we employed the partition-based graph abstraction (PAGA) algorithm to construct a cell fate map and infer transitional trajectories of these subsets. As illustrated in **Figures 5F, G**, each CD8+ T cell subset displayed a unique transitional trajectory to the other subsets. Notably, the CD8_Tem-GZMK subset was relatively closer to the CD8_Tex-LAYN subset compared to the CD8_Tn-LEF1 and CD8_Trm-KLRB1 subsets (**Figures 5F, G**), aligning with recent studies that highlight the transition from effector memory T cells to exhausted T cells during tumor progression. Interestingly, the CD8_Tn-LEF1 subset, representing the group of naïve T cells, was predicted to be located at the initial position (**Figures 5H**), suggesting a shared origin among these CD8+ T cell subsets in different states. Furthermore, we utilized the Single-Cell rEgulatory Network Inference and Clustering (SCENIC) (43) approach to analyze the activities of transcription factors (TFs) in the different CD8+ T cell subsets. Consistent with marker gene expression, these subsets also demonstrated distinct patterns of TF activity (**Figures 5I, J**), emphasizing the critical role of TFs in T cell differentiation. Overall, these findings highlight the differentiation of CD8+ T cells in LUAD patients, showcasing distinct patterns of gene expression and TF activity. This diversity leads to a range of cell states and functions during tumor progression and metastasis, underscoring the complex dynamics of CD8+ T cell subsets in the context of LUAD (44, 45).

### 3.6 Development of a risk assessment model using CD8_Tex-LAYN signatures in LUAD

**Figure 6:**
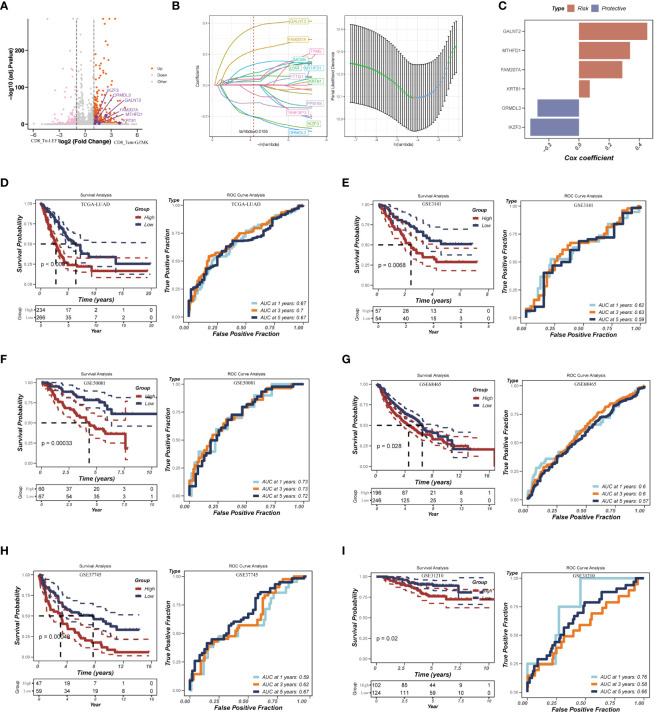
Construction of the risk assessment model with CD8_Tex-LAYN signatures. **(A)** Volcano plot for expression of differentially expressed genes between CD8_Tn-LEF1 and CD8_Tex-LAYN cells. The six risk genes used to construct model were highlighted. **(B)** Plots of each independent variable (left) and coefficient distributions for parameter selection (right). **(C)** The multivariate Cox coefficients for six risk genes. **(D-I)** Kaplan–Meier curves (left in each panel) and ROC curves (right in each panel) for TCGA and GEO LUAD cohorts.

Considering the significant enrichment of CD8_Tex-LAYN (exhausted CD8+ T cells) in tLung and mLN (**Figures 2D, E**), our next objective was to explore the potential of utilizing signatures within exhausted CD8+ T cells to accurately assess the risk of LUAD patients and develop appropriate treatment plans. To identify relevant signatures for risk assessment, we conducted differential expression gene (DEG) analysis and identified 487 genes specifically upregulated in CD8_Tex-LAYN (**Figure 6A**), which are likely associated with the progression of CD8+ T cell exhaustion. To streamline the selection of signatures, we employed Lasso Cox regression analysis, as previously reported (46), resulting in the identification of 12 candidate genes (**Figure 6B**, left) with lambda = 0.0155, indicating the lowest partial likelihood deviance (**Figure 3B**, right). Subsequently, using multivariate Cox regression with a stepwise regression method, we further narrowed down the selection to six final risk signatures: polypeptide N-acetylgalactosaminyltransferase 2 (GALNT2), methylenetetrahydrofolate dehydrogenase, cyclohydrolase, and formyltetrahydrofolate synthetase 1 (MTHFD1), family with sequence similarity 207 member A (FAM207A), keratin 81 (KRT81), ORMDL sphingolipid biosynthesis regulator 3 (ORMDL3), and IKAROS family zinc finger 3 (IKZF3). These six risk signatures were incorporated into a formula for classifying LUAD patients, as follows: RiskScore = 0.454 * GALNT2 + 0.339 * MTHFD1 + 0.288 * FAM207A + 0.071 * KRT81 - 0.276 * ORMDL3 - 0.323 * IKZF3 (**Figure 6C**).

To assess the accuracy of our risk assessment model, we applied the RiskScore calculation to a cohort of LUAD bulk RNA-seq samples obtained from TCGA database. After Z-mean normalization, the samples were divided into two distinct groups: high-risk (n = 234) and low-risk (n = 266). Importantly, the high-risk group exhibited significantly poorer survival outcomes (p< 0.0001) (**Figure 6D**, left). Moreover, the model demonstrated promising predictive performance, with AUC values of 0.67, 0.70, and 0.67 for 1-year, 3-year, and 5-year survival, respectively (**Figure 6D**, right). To further validate the robustness of our model, we subjected it to testing using five additional GEO datasets (**Figures 6E-I**). The consistent results across these datasets further confirm the reliability and effectiveness of our approach. Overall, by identifying signatures in CD8_Tex-LAYN cells, we have successfully established an accurate and reliable model for the risk assessment of LUAD patients, highlighting the strong association between exhausted CD8+ T cells and poor prognosis in LUAD.

### 3.7 Nomogram development

**Figure 7:**
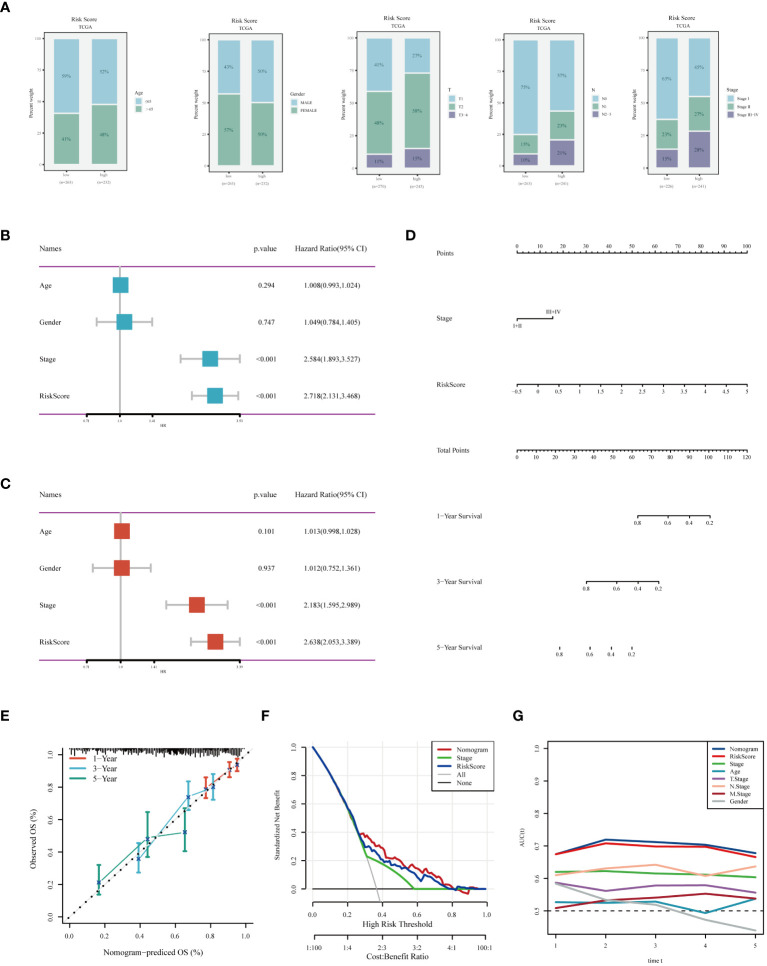
Analysis of Clinical Characteristics and Risk Evaluation in the TCGA-LUAD Dataset. **(A)** Analysis of the distribution of different clinical attributes across various risk groups. **(B)** Univariate Cox regression results indicating the relationship of clinical features with survival outcomes. **(C)** Evaluation of the independent prognostic significance of clinical factors using multivariate Cox regression. **(D)** A nomogram was developed by integrating clinical characteristics with the risk score. **(E)** Calibration plot for checking the accuracy of the prognostic model. **(F)** Decision curve analysis to determine the clinical utility of the prognostic model. **(G)** ROC curve analysis for assessing the predictive accuracy of the model over different time points.

We assessed the differences in clinical characteristics between patients stratified by different risk levels, finding that those in the low-risk group were younger, with a higher proportion of females, and typically presented with earlier pathological stages (**Figure 7A**). Both univariate and multivariate Cox analyses indicated that pathological stage and risk score are independent risk factors for LUAD prognosis (**Figures 7B, C**). Subsequently, we constructed a nomogram for predicting the prognosis of LUAD (**Figure 7D**). Calibration and decision curves demonstrated the robustness and accuracy of the nomogram (**Figures 7E, F**). The ROC curve highlighted the superior predictive performance of the nomogram over other features(**Figure 7G**).

### 3.8 Mutation analysis

**Figure 8:**
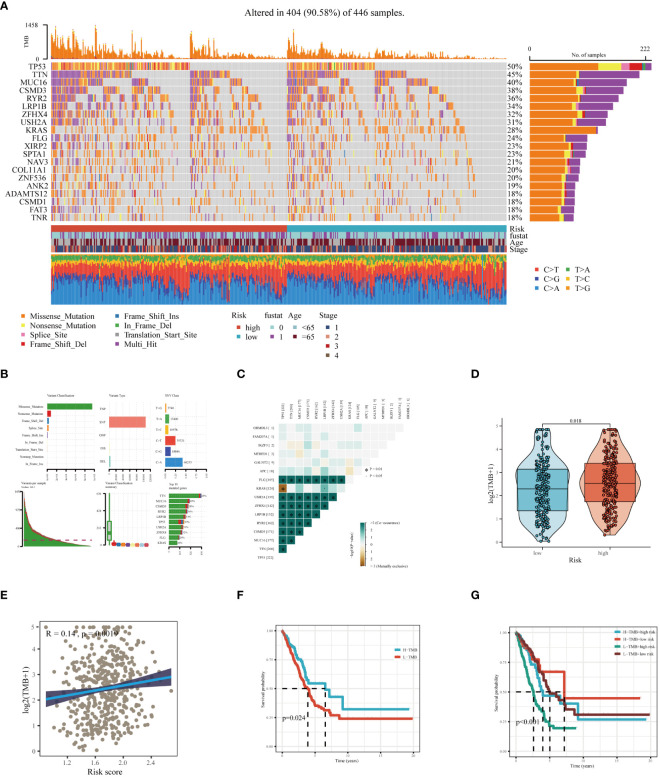
Mutation landscape analysis in LUAD. **(A)** Analysis of the mutation spectrum in high and low risk categories, highlighting the top 20 genes with the highest mutation rates in the TCGA-LUAD dataset. **(B)** Examination of mutation details in patients from the TCGA-LUAD study. **(C)** Interaction patterns between model genes and those frequently mutated. **(D)** Comparative assessment of Tumor Mutation Burden (TMB) across varying risk groups. **(E)** Investigation of the relationship between the risk score and TMB. **(F)** Kaplan-Meier curves depicting survival outcomes for groups with high and low TMB. **(G)** Combined multivariate Kaplan-Meier survival analysis using both TMB and risk score.

Through an extensive mutation analysis of the TCGA-LUAD dataset, we detailed the mutational profile of lung adenocarcinoma, identifying the 20 most commonly mutated genes and correlating them with clinical data across both high and low-risk patient groups (**Figure 8A**). As shown in **Figure 8B**, in TCGA-LUAD patients, the most common Variant Classification was Missense Mutation, the prevalent Variant Type was SNP, and the most frequent SNV Class was C>A. Subsequent co-mutation analysis did not reveal significant co-mutations between model genes and the most commonly mutated genes (**Figure 8C**). The high-risk group exhibited a higher TMB (**Figure 8D**), and there was a significant positive correlation between risk score and TMB (**Figure 8E**). Theoretically, tumors with a higher TMB generate a greater number of neoantigens, which may be recognized as foreign substances by the patient’s immune system, thereby triggering an attack on the tumor. Survival analysis of patients with high and low TMB demonstrated better prognosis in the high TMB group (**Figure 8F**). When combining TMB with risk score for survival analysis, patients in the low-TMB and high-risk category showed the poorest prognosis, while those in the high-TMB and low-risk category had a better prognosis (**Figure 8G**). In summary, these findings highlight the intricate relationship between genetic mutations, TMB, and patient prognosis in LUAD, underscoring the importance of comprehensive genomic profiling in risk assessment and treatment stratification.

### 3.9 Enrichment analysis

Our study comprehensively analyzed the relationship between risk scores, tumor-related pathways, and the tumor immune cycle. Findings revealed that risk scores were significantly positively correlated with most tumor-related pathways, yet exhibited a negative correlation with the majority of the tumor immune cycle stages (**Supplementary Figure 2A**). Further Gene Set Variation Analysis (GSVA) enrichment analysis indicated that high-risk groups were primarily associated with pathways like MYC_TARGETS_V2, GLYCOLYSIS, and MYC_TARGETS_V1 (**Supplementary Figure 2B**). GSEA conducted separately for high and low-risk groups showed significant enrichment of the high-risk group in pathways such as KEGG_CELL_CYCLE and DNA_REPLICATION, whereas the low-risk group was mainly enriched in KEGG_ALLOGRAFT_REJECTION and KEGG_INTESTINAL_IMMUNE_NETWORK_FOR_ICA pathways (**Supplementary Figures 2C, D**). In summary, these insights reveal a close link between risk scores and tumor biological characteristics, highlighting molecular pathway activity differences in patients with varying risk levels and providing an important perspective for understanding tumor development mechanisms.

### 3.10 Assessment of the tumor microenvironment

Utilizing seven algorithms - TIMER, CIBERSORT, CIBERSORT-ABS, QUANTISEQ, MCPCOUNTER, XCELL, and EPIC, we thoroughly evaluated the differences in immune cell infiltration levels between high and low-risk groups (**Supplementary Figure 3A**). Subsequent radar charts highlighted the disparities in immune-related functions and immune cell infiltration (**Supplementary Figures 3B, C**). Overall, the low-risk group exhibited more active immune-related functions and higher levels of immune infiltration. Additionally, using the ‘estimate’ R package, we assessed the differences in tumor purity between the high and low-risk groups. The results indicated that the high-risk group had higher tumor purity, and a significant positive correlation was observed between risk scores and tumor purity (**Supplementary Figure 3D**).

### 3.11 Immunotherapy response evaluation

**Figure 9:**
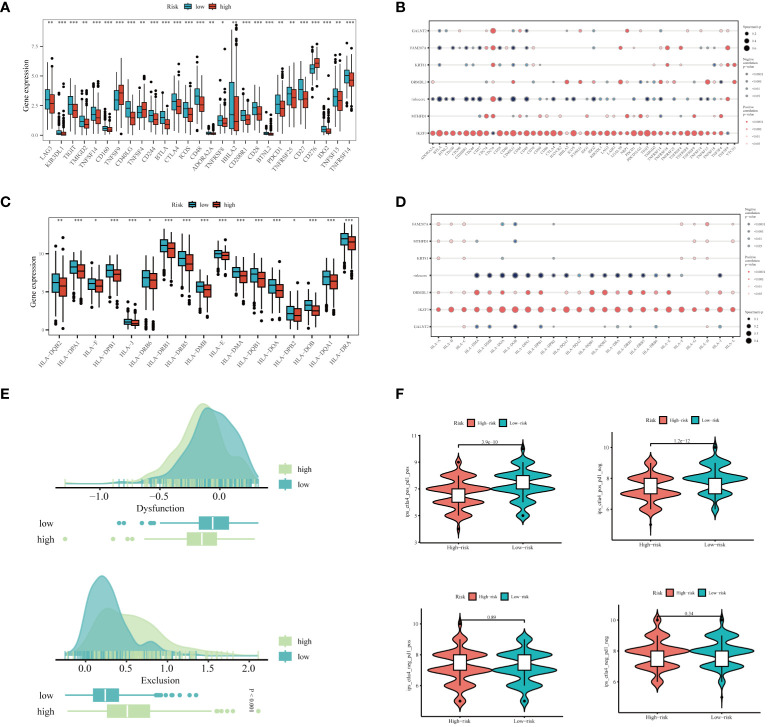
Prediction of Immunotherapy Efficacy. **(A)** Differences in immune checkpoint gene expression between high and low-risk groups. **(B)** Correlations between immune checkpoint gene expression, risk scores, and Hub genes. **(C)** Expression differences of major histocompatibility complex (MHC) genes between risk groups. **(D)** Correlations of MHC-related genes and immune checkpoint genes with risk scores and Hub genes. **(E)** Differences in TIDE scores across the high and low-risk categories. **(F)** Comparative Immune Predictive Scores (IPS) between high and low-risk groups. *P < 0.05, **P < 0.01, ***P < 0.001.

In light of the substantial advancements in immunotherapy for LUAD, we aimed to assess the differential responsiveness to immunotherapy between high and low-risk groups through a series of analyses. Initially, patients in the low-risk group exhibited higher expression levels of immune checkpoint-related genes (**Figure 9A**). There was a tendency for the risk score to be inversely correlated with these genes, interestingly, IKZF3 showed a significant positive correlation with them (**Figure 9B**). Similarly, the expression levels of MHC-related genes were significantly higher in the low-risk group compared to the high-risk group (**Figure 9C**), with an inverse correlation observed between risk score and MHC-related genes (**Figure 9D**). The detailed list of the genes mentioned can be found in **Supplementary Table 2**. Based on the TIDE database, patients in the high-risk group were found to be more prone to immune escape (**Figure 9E**). Subsequent analysis of Immunophenoscoring (IPS) differences revealed that patients in the low-risk group, especially those positive for CTLA4, are more likely to benefit from immunotherapy (**Figure 9F**). Overall, our multi-faceted evaluation of immunotherapy sensitivity across different groups demonstrated that the low-risk group not only expresses higher levels of immune checkpoint and MHC-related genes but is also more likely to benefit from immunotherapy, particularly in CTLA4 positive patients.

### 3.12 Experimental validation *in vitro*

**Figure 10:**
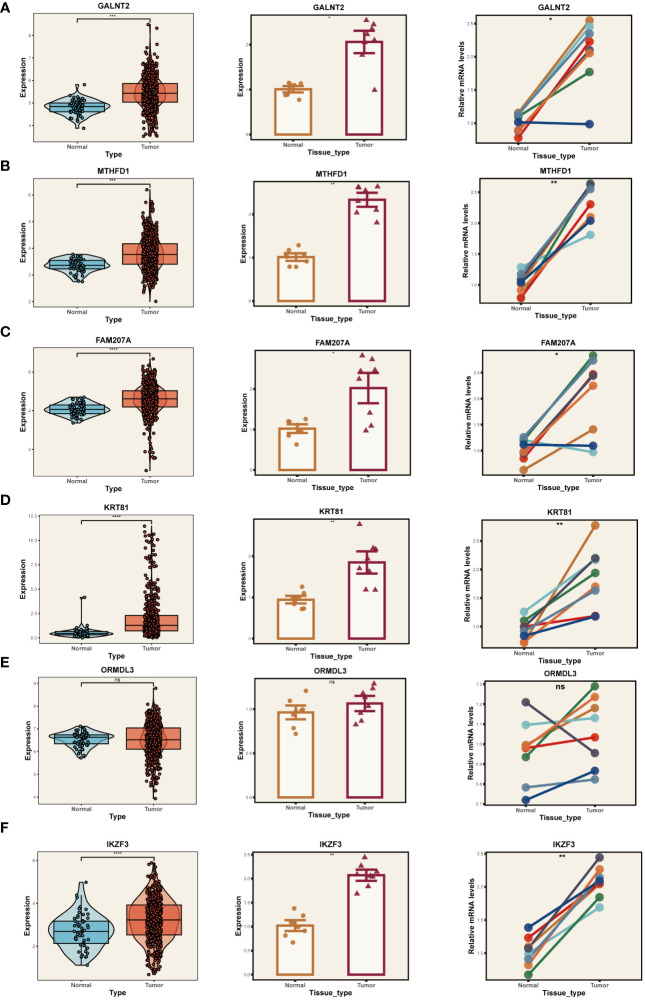
PCR Validation of Gene Expression. Expression of genes GALNT2 **(A)**, MTHFD1 **(B)**, FAM207A **(C)**, KRT81 **(D)**, ORMDL3 **(E)**, and IKZF3 **(F)** in tumor and normal tissue samples. Left panels: Distribution of expression in the TCGA database. Middle panels: Expression in tumor vs. normal tissue from Tianjin Chest Hospital. Right panels: Paired comparison between individual tumor and adjacent normal tissues. *P < 0.05, **P < 0.01, ***P < 0.001.

Given the reliance of our analysis on publicly available databases, we undertook a series of fundamental experiments to validate the bioinformatics findings. Initially, we collected tumor and adjacent normal tissue samples from eight LUAD patients who underwent surgical resection at Tianjin Chest Hospital. RT-PCR was employed to validate the expression of model genes (**Figures 10A-F**). Our results indicated significant overexpression of GALNT2, MTHFD1, FAM207A, KRT81, and IKZF3 in tumor tissues, consistent with our bioinformatics analysis. No expression difference was noted for ORMDL3 between tumor and adjacent tissues.

**Figure 11:**
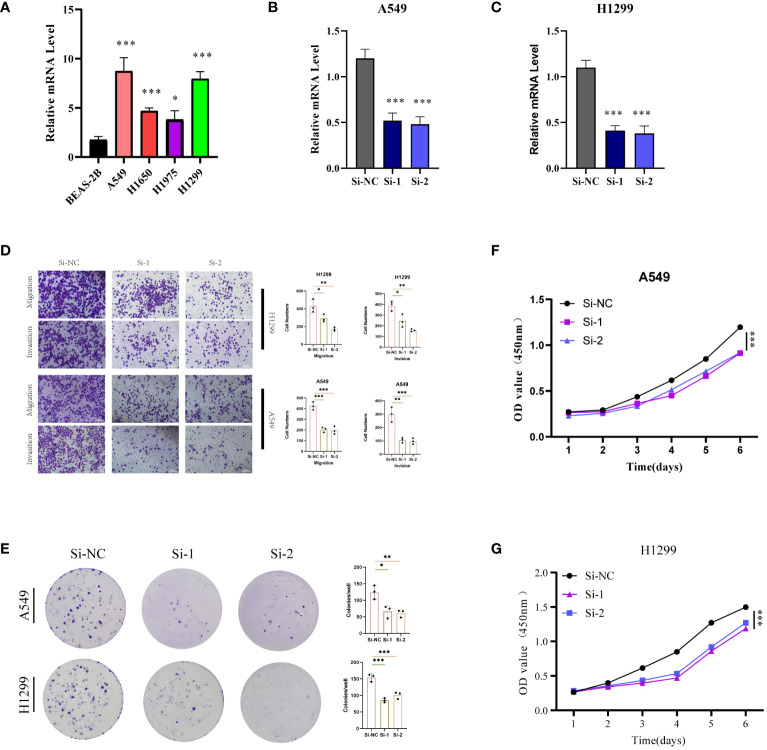
Experimental Validation *In Vitro*. **(A)** RT-PCR validation of GALNT2 expression across five cell lines. Post-transfection (48 hours), RT-PCR was utilized to confirm GALNT2 RNA expression levels in **(B)** A549 and **(C)** H1299 cell lines. **(D)** Transwell assays were conducted to evaluate the impact of GALNT2 knockdown on the migratory and invasive capabilities of A549 and H1299 cells. **(E)** Colony formation assays were performed to assess the proliferation potential of A549 and H1299 cells post GALNT2 knockdown. CCK-8 assays detected the effects of GALNT2 knockdown on cell proliferation abilities in **(F)** A549 and **(G)** H1299 cell lines. *P < 0.05, **P < 0.01, ***P < 0.001.

With the highest risk coefficient in the risk model (0.454), GALNT2 was further investigated for its role in LUAD using various cellular assays. Expression levels of GALNT2 were first examined across normal pulmonary epithelial (BEAS-2B) and 4 LUAD cell lines (A549, H1299, H1975, H1299) (**Figure 11A**), revealing a significant upregulation in LUAD cells, particularly in A549 and H1299. Post-transfection RT-PCR confirmed the knockdown efficiency (**Figures 11B, C**). Transwell assays demonstrated that GALNT2 knockdown notably inhibited the migratory and proliferative abilities of LUAD cells (**Figure 11D**). Colony formation assays showed a reduction in colony numbers post GALNT2 knockdown (**Figure 11E**). Finally, CCK8 assays confirmed that knocking down GALNT2 significantly suppressed the proliferation of LUAD cell lines (**Figures 11F–G**). In summary, our experimental validation reinforces the bioinformatics predictions by confirming the overexpression of several risk genes, including GALNT2, in LUAD tissues compared to adjacent non-tumor tissues. The significant inhibitory effects on migration, proliferation, and colony formation in LUAD cell lines upon GALNT2 knockdown highlight its potential role as a therapeutic target.

## 4 Discussion

This study aimed to construct a comprehensive single-cell atlas of LUAD through scRNA-seq of 29 samples from 19 treatment-naïve LUAD patients. The atlas detailed cellular compositions and functionalities within the LUAD microenvironment, particularly focusing on epithelial cells, stromal cells (including fibroblasts and endothelial cells), and a variety of immune cells (comprising T cells, B cells, myeloid cells, and NK cells). By identifying seven major cell lineages and delving into the immune cells, we elucidated their collaborative roles in tumor progression and immune response regulation within TME. Furthermore, leveraging marker genes associated with exhausted CD8+ T cells, we established a robust prognostic model for stratifying LUAD patient risk. This model facilitated a systematic assessment of differences in prognosis, TME, mutation landscape, and immune therapy responses across varied risk groups. Our findings not only deepen the understanding of tumor immunology in LUAD but also provide new directions for future therapeutic strategies and improvement of patient prognosis.

In this study, we elucidated the integral roles of DC1, DC2, and pDC within the MHC-II signaling pathway in the context of the LUAD microenvironment. It was observed that MHC-II, predominantly expressed on antigen-presenting cells, is also present in cancer cells, potentially correlating with improved immunotherapy outcomes (47). Evidence from recent studies suggests a positive correlation between MHC-II expression in tumor cells and the effectiveness of immunotherapeutic interventions, underscoring its pivotal role in tumor immunology (48). Tumor cells expressing MHC-II potentially secrete immunostimulatory exosomes, engage in direct interactions with CD4+ T cells to influence their polarization and activation, or secrete antigens that are endocytosed and presented by professional antigen-presenting cells (pAPCs). These mechanisms collectively contribute to modulating the immune landscape within the tumor microenvironment. Adjusting MHC-II expression in melanoma may enhance responsiveness to immunotherapies (49). Furthermore, our investigation revealed dynamic interactions within the Macrophage Migration Inhibitory Factor (MIF) signaling pathway among myeloid cell subtypes, with a notable emphasis on the critical function of pDCs. The MIF pathway is essential in orchestrating the immune responses within the tumor milieu, especially in modulating cellular interactions and immune cell activity. Overexpression of MIF in various cancer types is associated with increased tumor aggressiveness and adverse prognostic outcomes (50). This study also highlights the role of MIF in cell survival via Akt pathway activation and the involvement of CSN5/JAB1 in the regulation of autocrine MIF activity (51).

Our scRNA-seq study revealed a dynamic shift in CD8+ T cells from a naïve to an exhausted phenotype as LUAD progresses, mirroring recent discoveries in other cancers such as breast and colorectal (40, 52). Based on the marker genes of exhausted CD8+ T cells, we constructed a risk model composed of 6 genes, namely *GALNT2, MTHFD1, FAM207A, KRT81, ORMDL3, IKZF3*. The function of *GALNT2* and a series of basic experimental validations will be discussed in the following sections. Next, we discuss the potential roles of the remaining five model genes in the progression of LUAD. *MTHFD1* encodes a protein that plays a critical role in the folate metabolic pathway, which is essential for DNA synthesis, repair, and methylation (53). Alterations in folate metabolism can contribute to carcinogenesis by affecting DNA methylation patterns and thus gene expression (54). In the context of LUAD, *MTHFD1* could influence tumor progression through effects on DNA synthesis and methylation, potentially affecting cell proliferation and survival (55). Given its classification as a risk signature gene, *FAM207A* may be involved in processes that promote tumor growth or metastasis, possibly through affecting cell adhesion, migration, or communication within the TME (56). *KRT81*, as a member of the keratin family, is involved in the structural integrity of epithelial cells (57). Keratins are often implicated in cancer through their roles in epithelial cell stability, migration, and invasion (58). *KRT81* could contribute to LUAD pathogenesis by affecting tumor cell mechanical properties, facilitating invasion and metastasis (58). *ORMDL3* is involved in sphingolipid metabolism, which has been linked to various cellular processes important in cancer, including cell growth, apoptosis, and response to therapy (59). *ORMDL3*’s role in modulating sphingolipid metabolism could influence the survival and proliferation of LUAD cells, as well as their sensitivity to chemotherapeutic agents (60). *IKZF3*, as a member of the IKAROS family of zinc-finger transcription factors, plays a role in lymphocyte differentiation and function (61). Its involvement in LUAD could be related to immune evasion mechanisms, where altered expression of *IKZF3* affects the immune microenvironment’s ability to recognize and eliminate tumor cells. Leveraging these signatures, a Cox regression model was formulated, demonstrating substantial predictive accuracy and consistency, corroborated by data from the TCGA and six additional GEO cohorts. Our findings emphasize that patients categorized in the high-risk group, based on these gene signatures, showed significantly worse outcomes, underscoring the critical role of these genes in the advancement of LUAD. In advancing our research, we have integrated risk scores with clinical features to construct a novel nomogram. The diagnostic efficacy of this nomogram was superior compared to other clinical characteristics, as evidenced by its ROC curve analysis. Mutation analysis revealed a higher mutation frequency in the high-risk group. Furthermore, patients with high TMB exhibited markedly better survival outcomes compared to those with low TMB. Kaplan-Meier analysis for various factors indicated that patients in the high-TMB and low-risk category had a more favorable prognosis. This comprehensive approach, combining genetic risk factors with clinical parameters, offers a more refined and predictive model for patient outcomes in LUAD, potentially guiding more personalized therapeutic strategies.

Through enrichment analysis, we established a link between risk scores and tumor-related pathways in LUAD. This revealed a positive correlation with tumor-promoting pathways and a negative one with tumor immune cycle stages, highlighting the complex interplay between genetic risk and tumor environment. Distinct molecular pathways were associated with high-risk (cell cycle and DNA replication) and low-risk (immune-related) groups, suggesting different tumor progression mechanisms. Additionally, higher tumor homogeneity in high-risk groups, potentially influencing invasiveness, was noted, with low-risk groups showing greater potential for immunotherapy response, particularly in CTLA4 positive cases. These findings are crucial for understanding LUAD and developing personalized, immunotherapy-focused treatment strategies.

Our study then shifted its focus to GALNT2, which emerged as the gene with the highest risk score. Historically identified as a member of the glycosyltransferase family, GALNT2 was found to modulate adipogenesis and insulin signaling in adipocytes, impacting metabolic processes associated with obesity and diabetes (62). Its importance also extends to tumor development and progression, emphasizing its role in both metabolic and oncological pathways (63). Elevated GALNT2 levels are linked to unfavorable outcomes in patients with glioblastoma multiforme (GBM). In a functional context, inhibiting GALNT2 disrupts the growth, self-renewal, and aggressive behavior of glioma stem-like cells, primarily by downregulating CD44 expression (64). GALNT2 has been shown to contribute to the enhanced aggressiveness of colorectal cancer cells, doing so in part by modulating the AXL pathway (65). In this research, RT-PCR analysis revealed a marked upregulation of GALNT2 in LUAD. Subsequent cellular assays linked GALNT2 with enhanced proliferation and migration in LUAD cells, indicating its potential as a viable target for LUAD treatment strategies.

Our focus was primarily on primary lung tumors and lymph node metastasis in LUAD, without delving into the diverse clinical stages of the disease. More research is needed to explore LUAD’s progression at various stages. Although we’ve pinpointed critical genes and pathways related to LUAD, further external validation with functional experiments is required for confirmation. Despite these limitations, our study significantly aids in understanding LUAD’s microenvironment and provides a solid risk assessment model. Identifying risk genes linked to LUAD could lead to novel treatments. Targeting these genes and pathways may enhance treatment outcomes for LUAD patients, and we anticipate that our findings will be valuable for personalized treatment decisions, thereby improving LUAD’s clinical management. For hereditary tumors or high-risk populations, based on our predictive model, we can assess the risk of developing LUAD, thereby enabling early screening and monitoring of high-risk groups. Additionally, we found that the low-risk group is more likely to benefit from immunotherapy, which can help determine the best combination and sequence of treatment drugs to maximize efficacy and prolong survival. At the same time, by avoiding ineffective treatment plans, personalized treatment plans can help reduce the waste of medical resources, thereby reducing treatment costs and improving the efficiency and quality of medical services.

## Data availability statement

The original contributions presented in the study are included in the article/**Supplementary Material**. Further inquiries can be directed to the corresponding authors.

## Ethics statement

The studies involving humans were approved by Ethics Committee of Tianjin Chest Hospital. The studies were conducted in accordance with the local legislation and institutional requirements. The participants provided their written informed consent to participate in this study.

## Author contributions

HZ: Writing – original draft. PZ: Writing – original draft. XL: Writing – original draft. LT: Writing – original draft. YW: Writing – original draft. XJ: Writing – original draft. KW: Writing – review & editing. XL: Writing – review & editing. DS: Writing – review & editing.
