# Supplementary material for: Integrative single-cell analysis of LUAD: elucidating immune cell dynamics and prognostic modeling based on exhausted CD8+ T cells
Source: Front Immunol. 2024 Mar 26;15:1366096. doi: 10.3389/fimmu.2024.1366096 (PMC11002145; doi:10.3389/fimmu.2024.1366096)
Supplement: Supplementary file 1 [file DataSheet_1.docx]

Supplementary Material

## 1 Supplementary Figures


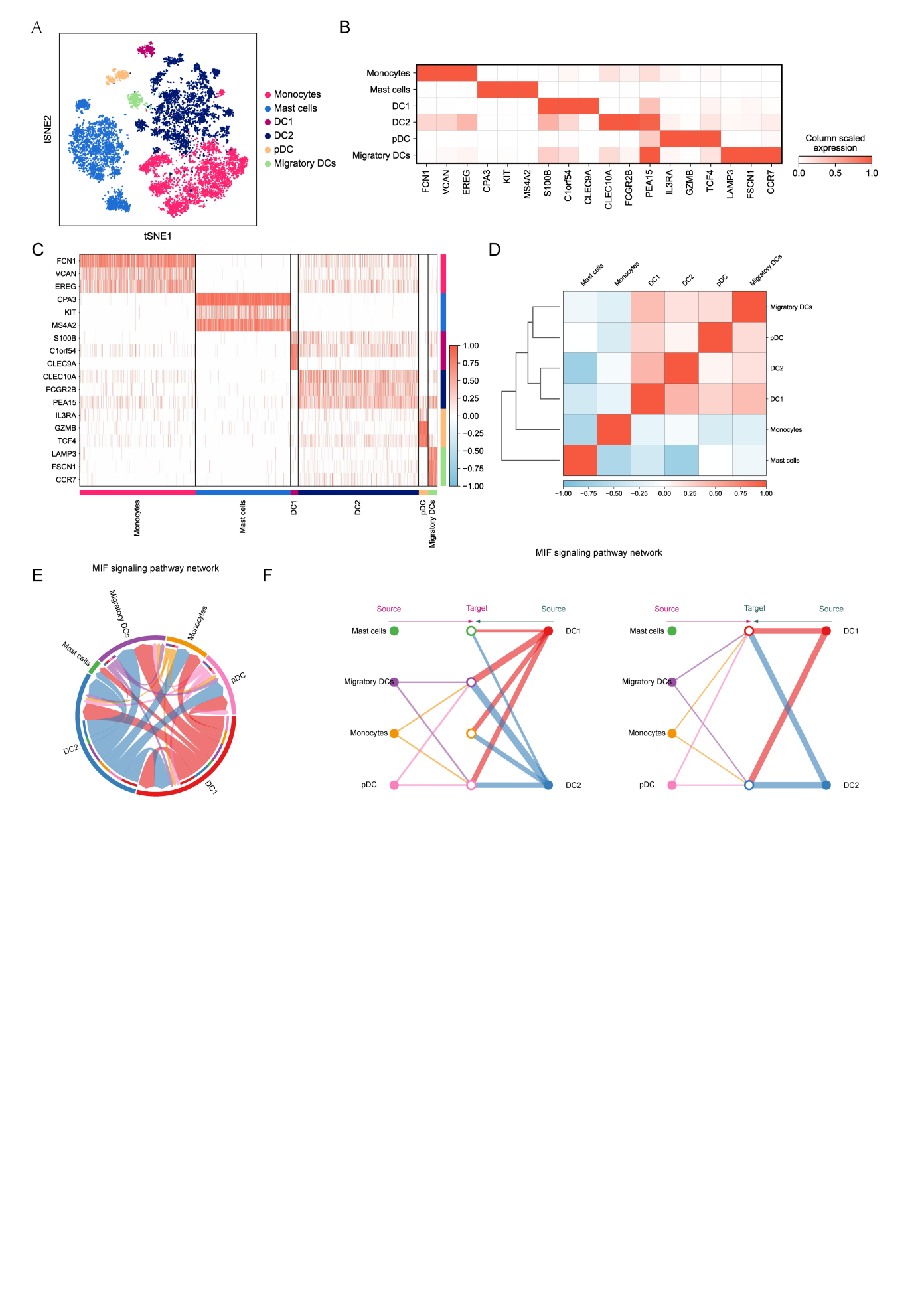


**Supplementary Figure 1 | Characterizing monocytes, mast cells and dendritic cells in LUAD.** (A) tSNE plot of 10,602 myeloid cells, including monocytes, mast cells and dendritic cells. (B, C) Dot plot and matrixplot of top3 DEGs in six myeloid cell subsets. (D) Correlations of gene expression among six myeloid cell subsets. (E) The inferred MIF signaling network by Cell-Chat. The edge width represents the communication probability. (F) Hierarchical plot showing the inferred intercellular communication network of MIF signaling pathway.


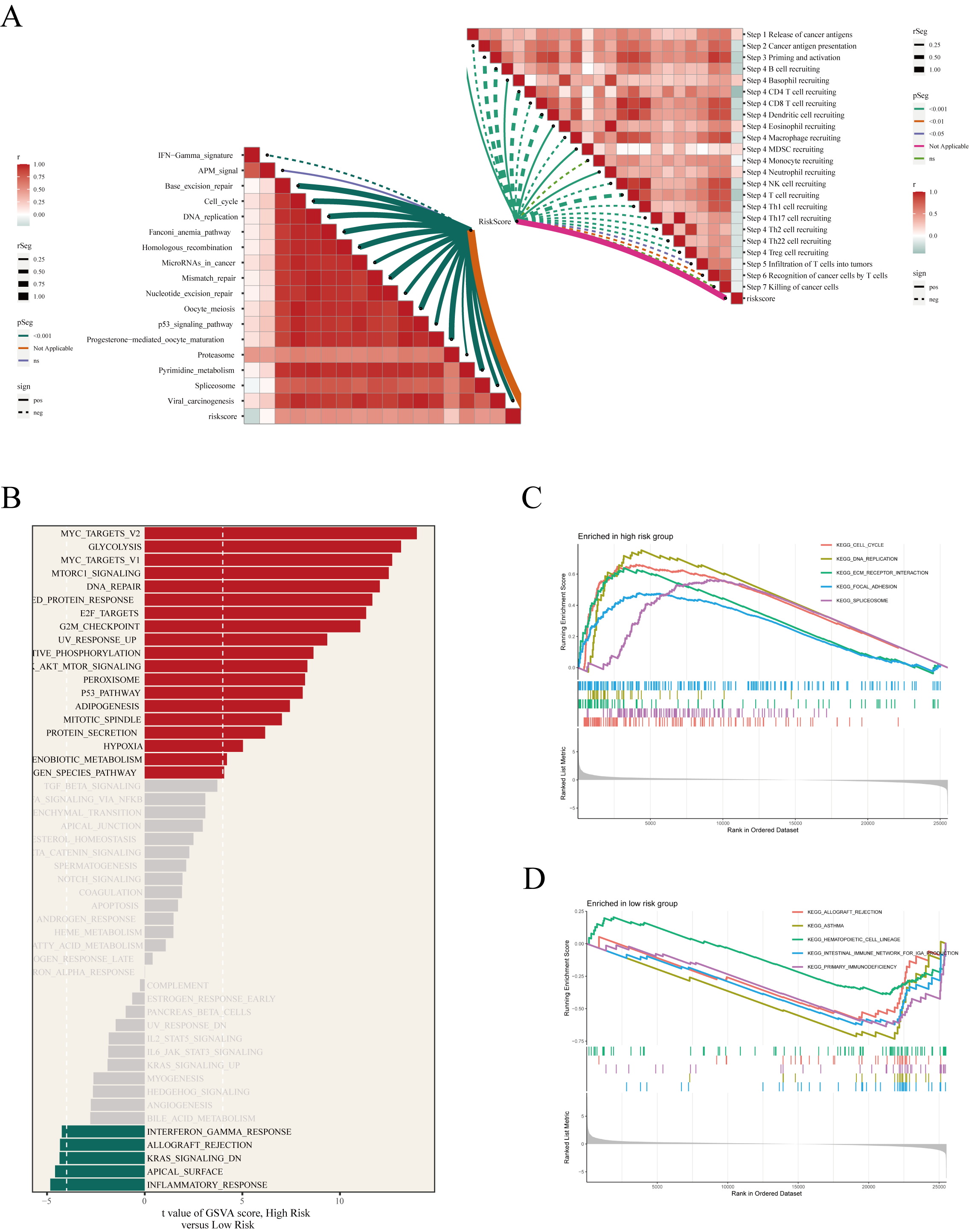


**Supplementary Figure 2 | Enrichment Analysis.** (A) Correlation between risk score and tumor immunity cycle and tumor-related pathways. The heatmap reveals the strength of the associations, with the color intensity indicating the level of correlation. (B) GSVA analysis showing significantly enriched pathways between high and low-risk groups. Bar plot illustrates pathways with higher enrichment scores in the high-risk group (red) versus the low-risk group (green). KEGG enrichment analysis results for patients in the (C) high-risk and (D) low-risk group. The line plot depicts the cumulative distribution of enrichment scores for selected pathways.


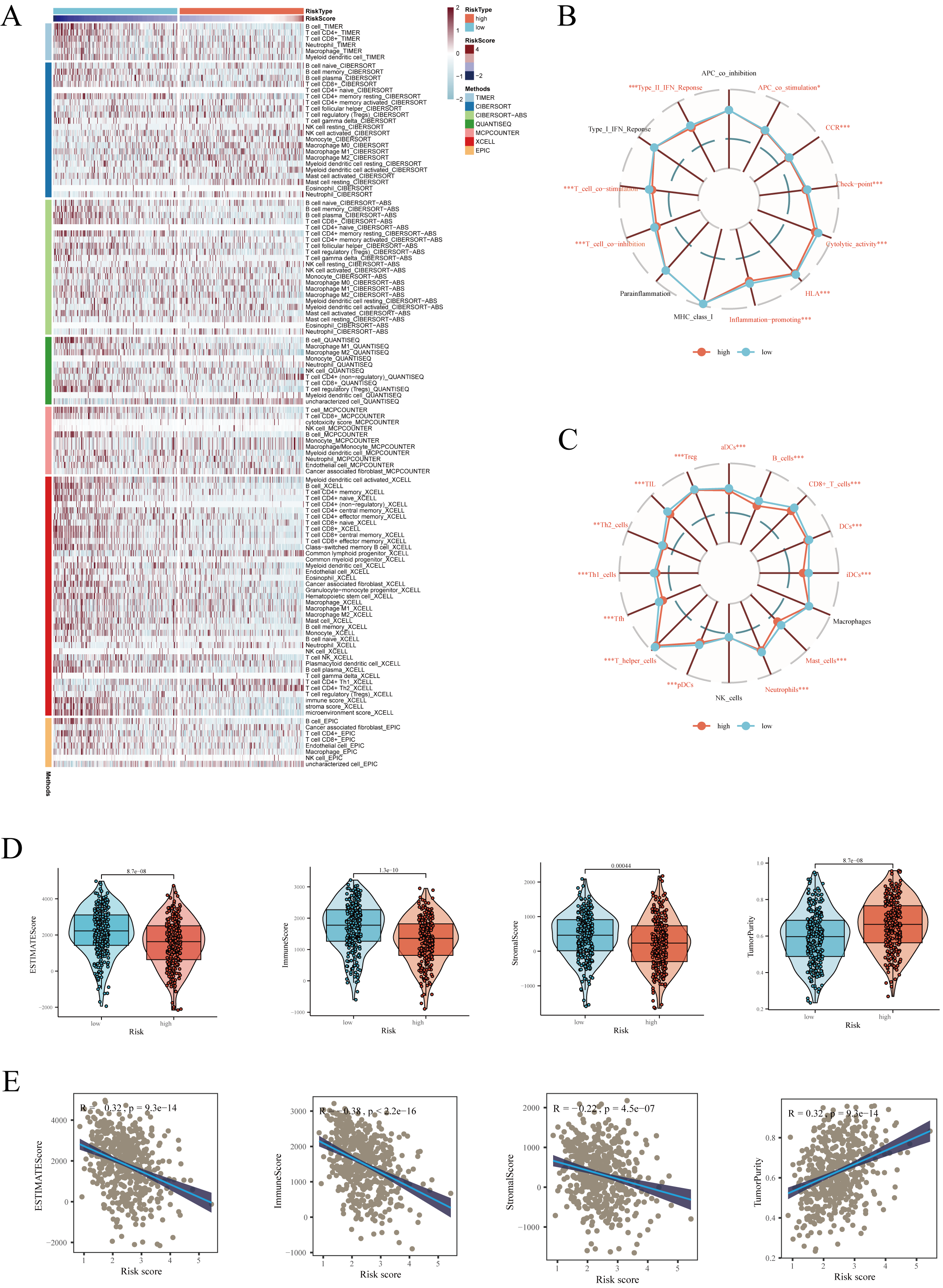


**Supplementary Figure 3 | Immune Infiltration Analysis.** (A) Assessment of immune cell infiltration levels in high vs. low-risk groups using seven algorithms. The heatmap illustrates the degree of infiltration by different immune cell types, ranging from low (blue) to high (red). (B) Evaluation of immune-related functions in different risk groups based on the ssGSEA algorithm. Radar charts display the expression levels of various immune functions across the two groups. (C) Differences in immune cell infiltration between risk groups. Radar charts show the relative abundance of specific immune cell types in different risk groups. (D) Differences in ESTIMATEScore, ImmuneScore, StromalScore, and TumorPurity between patients in high and low-risk groups. Violin plots depict the distribution of each score. (E) Correlation between risk score and ESTIMATEScore, ImmuneScore, StromalScore, and TumorPurity. Scatter plots demonstrate the associations between risk scores and these immune scores, with linear regression lines indicating trends.

|  |  | Primer sequences of model genes |
| --- | --- | --- |
| Name |  | Sequence (5'->3') |
| GALNT2 | Forward primer | CTGGGGAGTGGTAGAAGCAA |
|  | Reverse primer | AGACACTTGCGAATGGAGGA |
| MTHFD1 | Forward primer | AGCAGGGCTTTGGGAATCTC |
|  | Reverse primer | AAGATGGATGGTGATCGTTCCT |
| FAM207A | Forward primer | CGTGAGCAATGGTTGCAGAAA |
|  | Reverse primer | GCTTGTTGCTCTCCCTGC |
| KRT81 | Forward primer | GGGCTGTGAATGTCTGTGTCAG |
|  | Reverse primer | GTTCAATTGGCCGCAGGGC |
| ORMDL3 | Forward primer | CCAAACTCTGGATAAGGGGCA |
|  | Reverse primer | AACCCCAACTTAGGAGGACTCA |
| IKZF3 | Forward primer | AGAGCCTGAAATCCCTTACAGC |
|  | Reverse primer | GGAATGGGCGTTCACCAGTA |
| GAPDH | Forward primer | TGAACGGGAAGCTCACTGG |
|  | Reverse primer | TCCACCACCCTGTTGCTGTA |
|  |  |  |
|  |  | The sequences of siRNAs |
|  | Name | Sequences (5′-3′) |
|  | Si-NC | TTCTCCGAACGTGTCACGT |
|  | Si-GALNT2-1 | GGTGATCACGTTTCACAATGA |
|  | Si-GALNT2-2 | GGATGACTACAGCAATGATCC |

**Supplementary Table1 |** Primer Sequences for Model Genes and GALNT2 siRNA Sequences.

| checkpoint inhibitor-related genes | IDO1,LAG3,CTLA4,TNFRSF9,ICOS,CD80,PDCD1,LG2,TIGIT,CD70,TNFSF9,ICOSLG,KIR3DL1,CD86,PDCD1,LAIR1,TNFRSF8,TNFSF15,TNFRSF14,IDO2,CD276,CD40,TNFRSF4,TNFSF14,HHLA2,CD244,CD274,HAVCR2,CD27,BTLA,LGALS9,TMIGD2,CD28,CD48,TNFRSF25,CD40LG,ADORA2A,VTCN1,CD160,CD44,TNFSF18,TNFRSF18,BTNL2,C10orf54,CD200R1,TNFSF4,CD200,NRP1 |
| --- | --- |
| HLA-related genes | HLA-E,HLA-DPB2,HLA-C,HLA-J,HLA-DQB1,HLA-DQB2,HLA-DQA2,HLA-DQA1,HLA-A,HLA-DMA,HLA-DOB,HLA-DRB1,HLA-H,HLA-B,HLA-DRB5,HLA-DOA,HLA-DPB1,HLA-DRA,HLA-DRB6,HLA-L,HLA-F,HLA-G,HLA-DMB,HLA-DPA1 |

**Supplementary Table2 | Gene list for predicting the efficacy of immunotherapy.**
